# Iron sensing in plants

**DOI:** 10.3389/fpls.2023.1145510

**Published:** 2023-03-08

**Authors:** Isabel Cristina Vélez-Bermúdez, Wolfgang Schmidt

**Affiliations:** ^1^Institute of Plant and Microbial Biology, Academia Sinica, Taipei, Taiwan; ^2^Genome and Systems Biology Degree Program, Academia Sinica and National Taiwan University, Taipei, Taiwan

**Keywords:** iron uptake, iron homeostasis, plant immunity, signal transduction, nutrient sensors

## Abstract

The ease of accepting or donating electrons is the raison d’être for the pivotal role iron (Fe) plays in a multitude of vital processes. In the presence of oxygen, however, this very property promotes the formation of immobile Fe(III) oxyhydroxides in the soil, which limits the concentration of Fe that is available for uptake by plant roots to levels well below the plant’s demand. To adequately respond to a shortage (or, in the absence of oxygen, a possible surplus) in Fe supply, plants have to perceive and decode information on both external Fe levels and the internal Fe status. As a further challenge, such cues have to be translated into appropriate responses to satisfy (but not overload) the demand of sink (i.e., non-root) tissues. While this seems to be a straightforward task for evolution, the multitude of possible inputs into the Fe signaling circuitry suggests diversified sensing mechanisms that concertedly contribute to govern whole plant and cellular Fe homeostasis. Here, we review recent progress in elucidating early events in Fe sensing and signaling that steer downstream adaptive responses. The emerging picture suggests that Fe sensing is not a central event but occurs in distinct locations linked to distinct biotic and abiotic signaling networks that together tune Fe levels, Fe uptake, root growth, and immunity in an interwoven manner to orchestrate and prioritize multiple physiological readouts.

## Introduction

As a component of redox chains and cofactor of numerous enzymes, iron (Fe) is an indispensable micronutrient for all forms of life. In plants, Fe has essential functions in photosynthesis and is critical to chlorophyll biosynthesis. Iron is an abundant element in most soils; however, only a minor fraction of the total Fe is available for uptake by plant roots. This shortage is caused by the formation of Fe(III) oxyhydroxides with extremely low solubility, in particular in alkaline soils (Vélez-Bermúdez and Schmidt, 2022). Only in extremely acidic soils or in the absence of oxygen, Fe is available in concentrations that may exceed the requirement of plants and cause oxidative damage due to the formation of radical oxygen species.

To extract sufficient Fe from the soil solution, land plants have evolved a suite of mechanisms aimed at increasing its solubility. Two main Fe acquisition strategies - referred to as strategy I and strategy II - have been distinguished that separate dicotyledonous and non-grass monocotyledonous species from grasses (Poaceae) (Marschner and Römheld, 1986). In strategy I plants (i.e., *Arabidopsis* or tomato), soil pH governs the prioritization of specific processes within the repertoire of Fe mobilizing mechanisms. At acidic pH, Fe acquisition relies on a tripartite protein complex that mediates the acidification of the apoplast, the reductive splitting of Fe^3+^-chelates, and the uptake of the released Fe^2+^ ion. In the model plant *Arabidopsis thaliana*, this complex is comprised of the H^+^-ATPase AHA2 (Santi and Schmidt, 2009; Stéger and Palmgren, 2022), the oxidoreductase FRO2 (Robinson et al., 1999), and the Fe^2+^ transporter IRT1 (Eide et al., 1996; Vert et al., 2002). In addition, genes involved in the biosynthesis and export of the catechol sideretin, i.e., the oxygenases F6’H1 and S8H, the cytochrome P450 enzyme CYP82C4, and the transporter PDR9 are induced upon Fe starvation (Fourcroy et al., 2014; Schmid et al., 2014; Tsai et al., 2018). Fe^3+^-sideretin serves as a substrate for the FRO2/IRT1 Fe uptake module. At circumneutral and alkaline pH, FRO2 function is compromised (Susín et al., 1996), and Fe acquisition relies chiefly or entirely on catecholic coumarins such as esculetin and fraxetin that form stable complexes with Fe^3+^ at elevated pH. Under such conditions, the production of fraxetin is favored by increased expression of *S8H*, while the expression of *CYP82C4* and, thus, the hydroxylation of fraxetin to sideretin is repressed (Gautam et al., 2021; Tsai and Schmidt, 2021). Fraxetin either reduces Fe^3+^, which is then taken up by IRT1, or forms Fe^3+^-[fraxetin]_3_ complexes that are taken up as such *via* an as yet unknown transporter (Robe et al., 2021).

Grasses (i.e., rice or barley) employ high-affinity metal chelators of the mugineic acid family - referred to as phytosiderophores - to mobilize and take up Fe. Phytosiderophores are secreted *via* TOM1 (Nozoye et al., 2011), followed by uptake of the loaded Fe(III)-phytosiderophore complex by the high-affinity YS1 and YSL transporters, i.e., ZmYS1 in maize, OsYSL15 in rice, or HvYS1 in barley (Curie et al., 2001; Lee et al., 2009; Yamagata et al., 2022). Similar to strategy I plants, rice and possibly other grasses, possess homologs of IRT1 (Ishimaru et al., 2006) that serve in the uptake of (ferrous) Fe when Fe^2+^ is abundant, for example under waterlogged conditions.

## Hemerythrin: A ubiquitous component in Fe sensing

**Figure 1:**
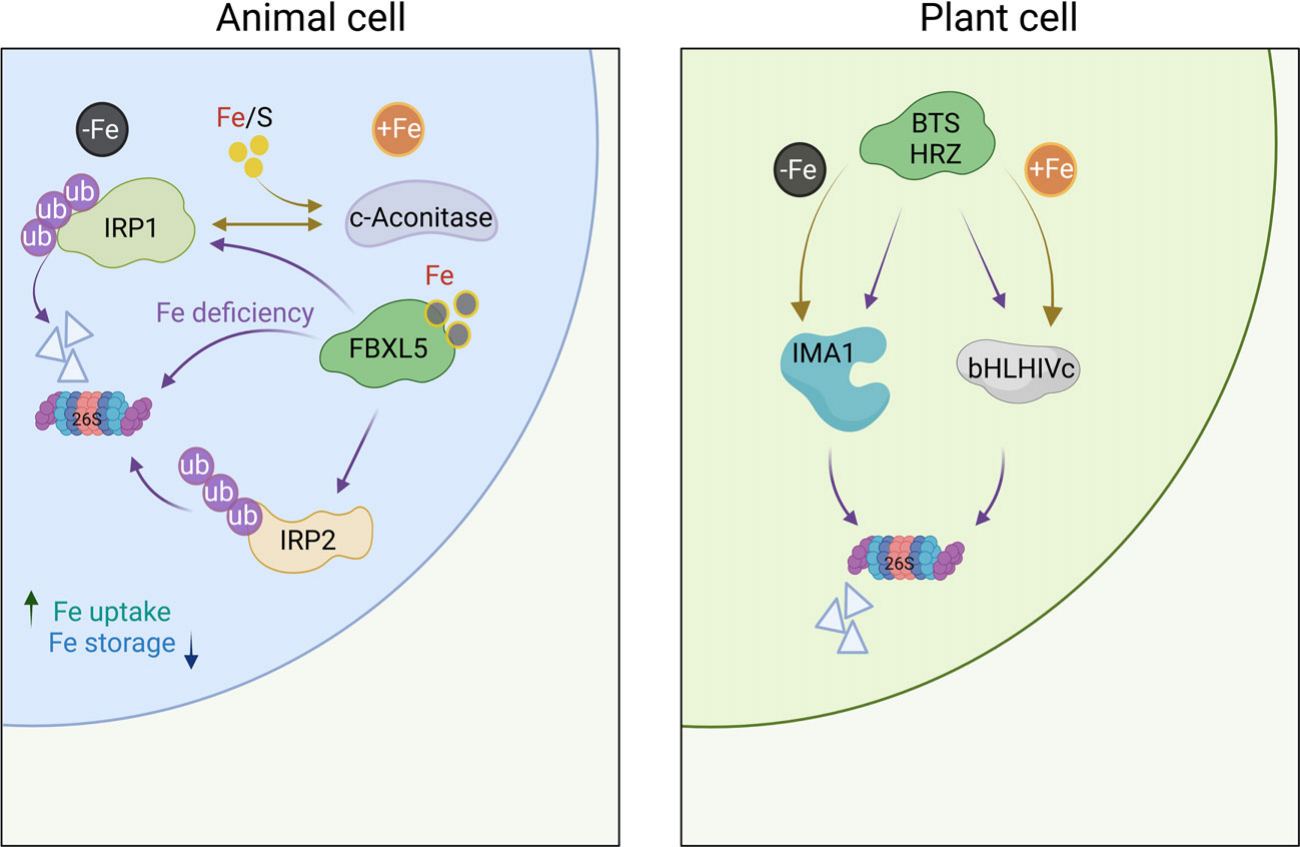
Iron sensing in mammals and plants. In mammalian cells, Fe is sensed *via* IRP1 and IRP2, which bind to IREs to regulate the translation of genes involved in the uptake and storage of Fe. In the presence of cytosolic Fe, IRP1 loses its sensing function and acts as an aconitase; both IRP1 and IRP2 are targeted for proteasomal degradation by FBLX5. In plants, homologs of FBLX5 (HRZ1/HRZ2) in rice and BTS/BTSL1/2 in Arabidopsis) regulate the activation of Fe-responsive genes *via* the abundance of clade IVc bHLH proteins, which are subjected to proteasomal degradation in the presence of Fe. Under Fe-deficient conditions, IMA/FEP peptides compete with clade IVc proteins for binding to HRZ/BTS, thereby revoking the inhibition of the Fe deficiency response by degrading IMA peptides instead of clade IVc bHLH proteins. Whether plants are able to sense Fe *via* a possible moonlighting function of cytosolic aconitase (ACO) enzymes has not been conclusively demonstrated for plants. Purple arrows indicate proteasomal degradation. Figure was created with BioRender.com.

How plant cells sense Fe remained opaque for a long time. In mammals, cellular Fe levels are monitored by the cytosolic Fe regulatory proteins IRP1 and IRP2. Under Fe-sufficient conditions, IRP1 is assembled with an [4Fe–4S] Fe–S cluster and acts as aconitase, converting citrate to isocitrate (**Figure 1**). Decreasing cellular Fe levels causes loss of the Fe-S cluster, which is essential for aconitase function. Freed from its metabolic duties, the apoprotein is able to bind to Fe-responsive elements (IREs) within the 5′ or 3′ UTR of mRNAs transcribed from genes encoding proteins involved in Fe uptake and sequestration (Rouault, 2006). Also IRP2 binds to IREs, but lacks aconitase activity and does not harbor an Fe-S cluster. IRP2 activity is, instead, dictated by ubiquitination and proteasomal degradation. The E3 ubiquitin ligase complex controlling IRP2 contains the F-box protein FBXL5, which harbors an Fe-binding hemerythrin domain. Iron deficiency destabilizes FBXL5, decreases E3 ubiquitin ligase activity, and increases IRP2 binding to IREs (Chollangi et al., 2012). Similar to IRP2, IRP1 can be targeted by FBXL5 for proteasomal degradation (Iwai, 2019). In plants, such a moonlighting function of cytosolic aconitase has been questioned by the observation that, in *Arabidopsis*, knockout of aconitase (ACO) genes had minor effects on the plant’s Fe status (Arnaud et al., 2007). In rice, however, defects in ACO1 lead to reduced expression of a subset of Fe-responsive genes, putatively by interaction of ACO with stem-loop structures of RNA molecules (**Figure 1**) (Senoura et al., 2020). The reasons for the differences between *Arabidopsis* and rice plants have not been conclusively addressed. Genetic redundancy among *ACO* genes in *Arabidopsis* might be causative for the incongruent results.

**Figure 2:**
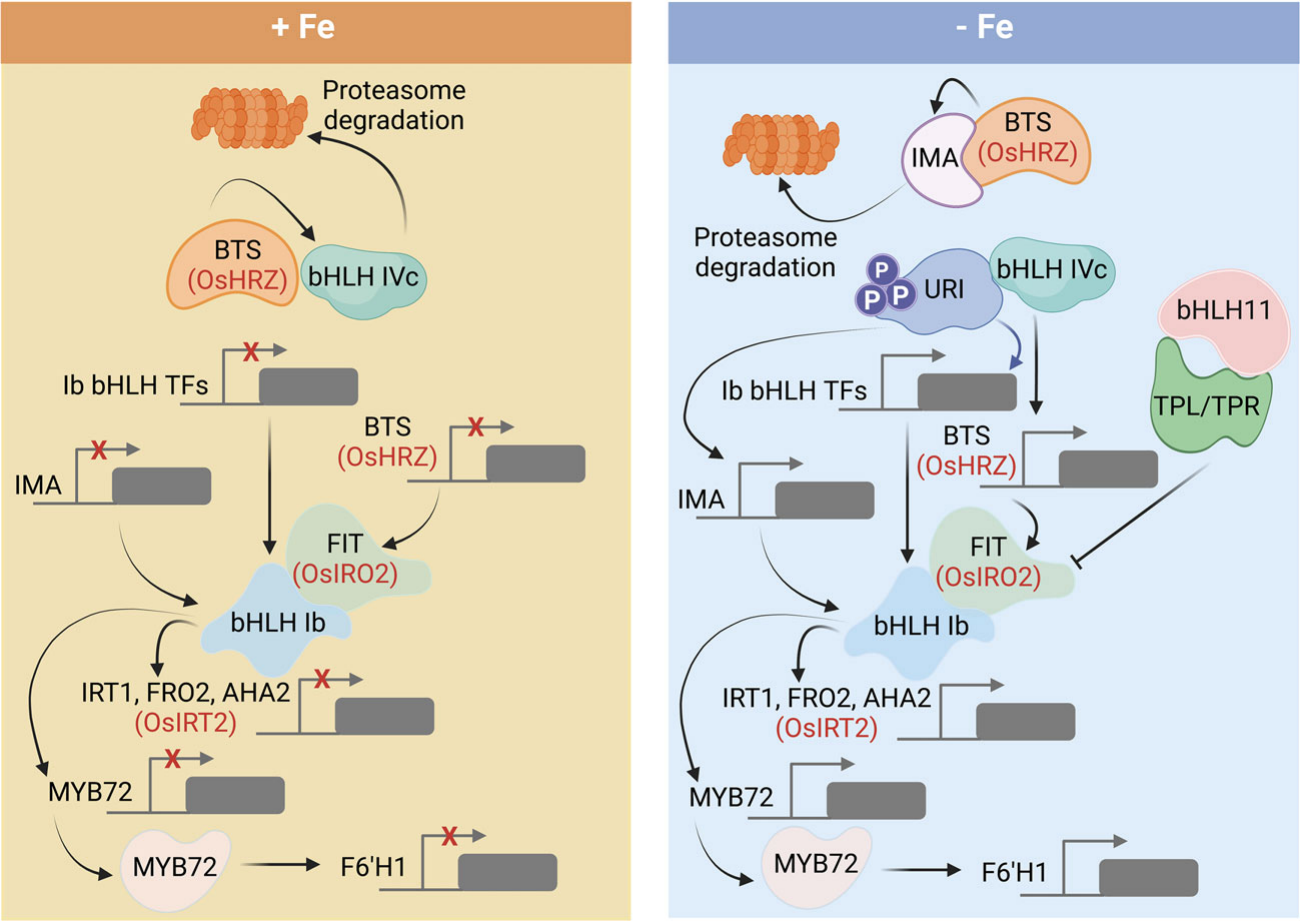
Regulatory cascade governing the expression of genes involved in Fe uptake and homeostasis in *Arabidopsis* and rice. Under Fe-sufficient conditions, BTS degrades clade IVc bHLH proteins and inhibits downstream signaling. When cytosolic Fe becomes limited, URI (clade IVb) is phosphorylated and promotes the transcription of clade Ib bHLH proteins, which forms heterodimers with FIT (bHLH29) to induce the root Fe uptake. bHLH11 recruits TOPLESS/TOPLESS-RELATED (TPL/TPRs) co-repressors and counteracts the expression of clade Ib bHLH transcription factors. Detailed explanations regarding the various components are given in the text. Rice homologs are denoted in red letters. Figure was created with BioRender.com.

While a possible function of plant aconitase in the perception of Fe awaits further clarification, solid support for a role in Fe sensing was provided for the hemerythrin domain-containing proteins HRZ1 and HRZ2 and their homologs in *Arabidopsis*, BRUTUS (BTS) and the BRUTUS LIKE proteins BTSL1/BTSL2 (Long et al., 2010; Kobayashi et al., 2013; Selote et al., 2015; Hindt et al., 2017; Aung et al., 2018; Rodríguez-Celma et al., 2019). *OsHRZ*-knockdown plants are more tolerant to Fe deficiency and display higher Fe levels in shoots and grains than the wild type, suggesting that OsHRZ negatively regulates Fe uptake (Kobayashi et al., 2013). In support of this supposition, *hrz* mutant lines display severe Fe toxicity symptoms when grown on high Fe media (Aung et al., 2018). The C-terminal regions of HRZ/BTS proteins harbor a RING domain that confers E3 ligase activity for ubiquitination, making these Fe sensors functionally similar to the mammalian Fe regulator FBXL5 (**Figure 1**). In contrast to FBXL5, which is stabilized by the presence of Fe, BTS and HRZ are unstable under Fe-sufficient conditions (Selote et al., 2015; Guo et al., 2021). In *Arabidopsis*, loss-of-function of BTS, BTSL1, or BTSL2 causes constitutive activation of Fe-responsive genes (Hindt et al., 2017; Rodríguez-Celma et al., 2019), suggesting functional homology to HRZ proteins. Under Fe-sufficient conditions, BTS and HRZ bind clade IVc bHLH proteins (bHLH105/ILR3, bHLH115, bHLH34/IDT1, and bHLH104 in *Arabidopsis*, and POSITIVE REGULATOR OF IRON DEFICIENCY RESPONSE (PRI) 1-4 in rice), and mediate their degradation (Selote et al., 2015; Xing et al., 2021). In the absence of Fe, clade IVc proteins are stable and promote a signal cascade that ultimately triggers root Fe uptake (**Figure 2**) (Gao et al., 2019; Akmakjian et al., 2021; Liang et al., 2020).

## Ironman and other superheroes: Fe sensing in plants

In *Arabidopsis*, BTS binds the clade IVc bHLH proteins bHLH105 and bHLH115 *via* a C-terminal domain (Li et al., 2021). A similar domain is also found at the C-terminus of IRONMAN (IMA)/FE-UPTAKE-INDUCING PEPTIDE (FEP) peptides (Li et al., 2021). IMA/FEP constitutes a ubiquitous family of peptides present in all flowering plants, including the basal angiosperm *Amborella trichopoda* (Grillet et al., 2018; Hirayama et al., 2018). The *A. thaliana* genome harbors eight *IMA* genes; only two homologs of this family were found in rice (Grillet et al., 2018). In *Arabidopsis*, overexpression of *IMA* leads to pronounced accumulation of Fe and Mn in all plant parts; octuple *ima8x* mutants display an extremely chlorotic phenotype. Owing to their increased abundance upon Fe deficiency, IMA peptides compete with clade IVc bHLH proteins for binding to BTS, preventing their degradation and promote induction of downstream Fe deficiency responses (**Figure 2**) (Li et al., 2021). Interaction with IMA peptides, specifically IMA1/FEP3, has also been demonstrated for BTSL proteins (Lichtblau et al., 2022), indicative of functional homology between BTS and BTSLs. Similar to what has been observed for *bts* mutants, loss of BTSL1 and BTSL2 function induces constitutive Fe deficiency responses (Rodríguez-Celma et al., 2019). BTSL1 and BTSL2 also interact with FIT to mediate its ubiquitination (Rodríguez-Celma et al., 2019). The reason for this duality of Fe sensors may lie in their different localization. While BTS localizes to the stele, BTSL proteins are primarily expressed in the epidermis and cortex cells, providing a first barrier against Fe overload, which would be detrimental to the cells (Rodríguez-Celma et al., 2019). Notably, no BTSL homologs have been identified in graminaceous species.

OsIMA1 and OsIMA2 appear to function similar to *Arabidopsis* IMA/FEP peptides (Kobayashi et al., 2021). Overexpression of both homologs conferred Fe deficiency tolerance to rice plants and led to the accumulation of Fe in all tissues, including seeds. Both OsIMA1 and OsIMA2 interact with OsHRZ protein in the same manner as AtIMA with AtBTS (Peng et al., 2022), suggesting that this modus operandi of Fe uptake regulation is common in flowering plants. Notably, OsHRZ and AtBTS promote the degradation of IMA peptides to decrease their presence when Fe becomes available (Li et al., 2021; Peng et al., 2022). IMA peptides can bind Fe through an aspartic acid-rich region and may get destabilized in the presence of Fe (Grillet et al., 2018). A tempting hypothesis was put forward by Peng et al. (2022). The authors suggested that Fe bound to IMA peptides is delivered to the hemerythrin domain of HRZ/BTS proteins to inactivate them, and, subsequently, induce Fe uptake.

## Events at or downstream of HRZ/BTS

Interestingly, BTS activity is not only controlled by IMA peptides and Fe. In *Arabidopsis*, the pathogen *Pseudomonas syringae* hijacks BTS to manipulate the availability of Fe in the apoplast of its host (Xing et al., 2021). In *Arabidopsis* mutant lines that are unable to adequately perceive the effector protein AvrRps4 secreted by the bacterium, the bacterial attack inactivates BTS. Capture of BTS suppresses the degradation of clade IVc bHLH proteins and increases host Fe levels. It appears that the C-terminus of AvrRps4 acts in a similar manner than IMA peptides to promote downstream Fe signaling (Xing et al., 2021).

Two glutaredoxins, OsGRX6 and OsGRX9, have been identified as OsHRZ-interacting proteins. In addition, OsGRX6 interacts with the basic leucine zipper transcription factor OsbZIP83 (Kobayashi et al., 2022). All three proteins are putative targets of OsHRZs for ubiquitination and 26S proteasome-dependent degradation. Overexpression of *OsbZIP83* affected internal Fe translocation and phytoalexin biosynthesis, suggesting multiple roles of this module acting downstream of the OsHRZ pathway. The function of the glutaredoxins is unclear at present, but a role in Fe-S cluster-mediated Fe signaling or utilization is a possible scenario, which may be conserved in other species such as *Arabidopsis* (Yu et al., 2017; Cheng et al., 2020). Another possible function of glutaredoxins is inter-organ signaling. Such function was previously demonstrated for the plant-specific class III glutaredoxin family peptides CEP DOWNSTREAM 1 (CEPD1) and CEPD2, which regulate the expression of the nitrate transporter NRT2.1 (Ohkubo et al., 2017; Ota et al., 2020). While this is an exciting possible role of glutaredoxins, their exact function in Fe sensing still awaits further clarification.

In addition to its role as a hub governing root Fe uptake, BTS seems to have functions that go beyond Fe sensing. In *Arabidopsis* leaves, BTS negatively regulates genes involved in energy metabolism, affecting in particular mitochondrial and plastidal genes (Choi et al., 2022). This is, although unexpected, not entirely surprising since, owing to the role of Fe in electron transport, cellular Fe homeostasis is tightly connected with energy metabolism.

Whether HRZ has similar functions remains to be elucidated. In rice, and possibly other strategy II plants, a group of proteins specific to grasses may be involved in Fe sensing. IDE-binding factor 1 (OsIDEF1) is a metal-binding protein that is critical for inducing the early Fe deficiency response in rice (Kobayashi et al., 2007). An exact placement of OsIDEF1 into the puzzle of Fe sensing and signaling is still pending.

## Signaling Fe deficiency: An infinite puzzle?

The transcription factor FIT plays a key role in governing Fe acquisition in *Arabidopsis* by forming heterodimers with the clade Ib bHLH proteins bHLH38, bHLH39, bHLH100, and bHLH101 that confer DNA binding and act together with FIT as key regulators for a large suite of Fe uptake genes (Jakoby et al., 2004; Colangelo and Guerinot, 2004; Yuan et al., 2008; Wang et al., 2013; Cai et al., 2022; Liang, 2022) (**Figure 2**). Induction of FIT/bHLH Ib is dependent on clade IVc bHLH proteins (Gao and Dubos, 2021). A further layer of regulation is added by the clade IVb proteins bHLH121 (UPSTREAM REGULATOR OF IRT1/URI), bHLH11, and bHLH47 (POPEYE/PYE) (**Figure 2**). Under Fe-deficient conditions, URI is phosphorylated and interacts with bHLH IVc proteins to induce the transcription of genes encoding clade Ib transcription factors (Kim et al., 2019; Gao et al., 2020; Lei et al., 2020). Binding of clade IVc proteins to bHLH11, on the other hand, represses the expression of clade Ib bHLH transcription factors by recruiting TOPLESS/TOPLESS-RELATED (TPL/TPRs) co-repressors (Li et al., 2022). PYE is another negative regulator of the Fe deficiency response (Long et al., 2010). PYE interacts with the bHLH IVc proteins bHLH104, ILR3/bHLH105, and bHLH115 to form a complex that represses *FERRITIN* and genes involved in Fe distribution (Tissot et al., 2019). Moreover, PYE can directly negatively regulate genes encoding clade Ib proteins as well as its own expression (Pu and Liang, 2022). Interestingly, IRL3 and PYE are critical for photoprotection by preventing the accumulation of singlet oxygen during Fe deficiency (Akmakjian et al., 2021).

A comparable regulatory module governs Fe uptake in rice plants (**Figure 2**). Here, the AtFIT homolog OsFIT/OsbHLH156 binds to the sole member of the rice bHLH Ib clade, IRON-RELATED TRANSCRIPTION FACTOR 2 (IRO2) (Ogo et al., 2007). The FIT-IRO2 heterodimer coordinates the expression of the rice Fe uptake genes IRT1, TOM1, YSL15, NAS1, NAS2, NAAT1, and DMAS1 (Liang et al., 2020; Wang et al., 2022). Similar to *Arabidopsis*, the OsFIT/OsIRO2 dimer is dependent on bHLH IVc and bHLH IVb proteins, which positively and negatively regulate downstream responses. PRI1-4 (clade IVc) proteins are critical for the induction of OsFIT/OsIRO2 (Kobayashi et al., 2019). OsIRO3 (clade IVb) is a homolog of AtPYE and acts as negative regulator of the Fe deficiency response (Zheng et al., 2010). Similar to *Arabidopsis* bHLH11 (but unlike PYE), IRO3 recruits TOPLESS/TOPLESS-RELATED (TPL/TPR) co-repressors and directly regulates *IRO2* expression (Li et al., 2022; Pu and Liang, 2022). IRO3 also binds to PRI1 and PRI2 to repress their transactivation activity (Li et al., 2022). The clade IVb protein bHLH61 binds to PRI1, and the bHLH61-PRI1 dimer recruits TPL/TPR to repress root-to-shoot translocation of Fe at high Fe levels (Wang et al., 2022).

## Multiple Fe sensors orchestrate plant responses to environmental cues

While Fe is only available for root cells exposed to the soil solution, the demand for the metal may differ dramatically among cell types and tissues and may be dynamically altered over time. In addition, pathogens may affect the requirement for Fe of sink tissues. Infection with the airborne necrotrophic fungus *Botrytis cinerea* was shown to activate the Fe deficiency response in *Arabidopsis*, probably caused by Fe depletion by the pathogen and defense responses based on the Fe-dependent generation of an oxidative burst (Lu and Liang, 2022). Iron deficiency induced by *B. cinerea* activated FIT-dependent signaling, which in turn triggered ethylene biosynthesis and ethylene-based immunity, suggesting overlapping Fe and immune signaling cascades.

A central node of convergence of distinct environmental signals is the R2R3-MYB-like transcription factor MYB72, first identified as a prerequisite for growth on alkaline substrates (Palmer et al., 2013). MYB72 regulates the secretion of Fe-mobilizing coumarins particularly at alkaline pH (Gautam et al., 2021), thereby enabling plants to extract Fe from recalcitrant pools and to thrive in calcareous soils. MYB72 was also identified as a key player in induced systemic resistance (ISR; Zamioudis et al., 2014). Conspicuously, both Fe deficiency and volatile organic compounds from ISR-inducing Pseudomonas bacteria can trigger *MYB72* expression, indicating that Fe acquisition can be increased independently of the availability of the nutrient (Zamioudis et al., 2015). Homozygous *myb72* mutants are unable to mount ISR against various pathogens including *B. cinerea*, suggesting that MYB72 is required for ISR against a wide range of pathogens (Trapet et al., 2021).

A sensor mediating the crosstalk between multiple signaling pathways was recently identified in a GWAS approach (Platre et al., 2022). Extracellular domains of the LRR receptor kinase SRF3 sense a signal that communicates the lack of Fe or flg22-mediated Fe decrease and regulate root growth and bacterially elicited immune responses. Homozygous *srf3* mutants resemble the excess Fe phenotype of *bts-1* and *opt3-2* mutants, suggesting that SRF3 negatively regulates Fe uptake. SRF3 is preferential expressed on the (bulk) plasma membrane and at the neck of plasmodesmata and becomes depleted in response to low Fe conditions. Elicitation of bacterial immune responses results in SRF3-dependent decrease of cellular Fe levels, leading to what is referred to as nutritional immunity in animals, i.e., the sequestration of Fe as a means to limit pathogenicity. Whether SRF3 is connected to other Fe sensing elements and, if so, how the information from different nexuses is orchestrated to tune Fe uptake by roots remains to be established.

## Untangling Fe sensing: Peeling the onion

The remarkable complexity and ever-increasing number of transcription factors and regulators involved in Fe signaling makes the elucidation of the mechanisms underlying the perception and communication of the plant’s Fe status a *quasi*-Sisyphean task. Moreover, recent findings support the involvement of additional regulatory layers such as microRNAs and epigenetic control in Fe signaling (Singh et al., 2021; Bakirbas and Walker, 2022; Du et al., 2022; Zhu et al., 2022), which need to be considered to generate a more complete picture of the events that are employed to control cellular Fe homeostasis. Commonly, changes in the Fe status do not occur as such but in combination with other cues such as pathogen attack, alterations in soil pH, or imbalances in ion availability or uptake. Thus, the recalibration of the plant’s Fe status may involve prioritization of competing interests represented by source (roots) and sink (non-root) tissues, microbial Fe piracy, and the influence of beneficial bacteria, which may not act entirely selfless when hijacking the plant's Fe acquisition repertoire. Improper intracellular transport of Fe to sites of high demand such as mitochondria and chloroplasts constitutes a further factor that can affect Fe sensing. While *sensu strictu* an Fe sensor is defined by its ability to interpret the prevailing Fe level by binding or interacting with Fe, input derived from other stimuli may, more indirectly, affect downstream signaling that leads to readjustment of cellular Fe levels. It appears clear that neither decoding the exact mechanisms by which Fe is sensed nor solving the puzzle as to how this information is conveyed and interlinked with the perception and signaling of other cues has yet been completed. However, some milestones have been localized that may serve as a point of reference during the mapping of the circuits that secures plant fitness in the not always friendly arms race for Fe.

## Author contributions

IV-B and WS performed the literature search and wrote the manuscript. WS suggested the concept of the review. IV-B drafted the figures. All authors contributed to the article and approved the submitted version.
